# A review on hepatocyte nuclear factor-1beta and tumor

**DOI:** 10.1186/s13578-015-0049-3

**Published:** 2015-10-13

**Authors:** Dan-Dan Yu, Shi-Wei Guo, Ying-Ying Jing, Yu-Long Dong, Li-Xin Wei

**Affiliations:** Tumor Immunology and Gene Therapy Center, Eastern Hepatobiliary Surgery Hospital, The Second Military Medical University, 225 Changhai Road, 200438 Shanghai, China

**Keywords:** Hepatocyte nuclear factor-1beta (HNF1β), Cancer, Stem/progenitor cells, Pathogenesis

## Abstract

Hepatocyte nuclear factor-1beta (HNF1β) was initially identified as a liver-specific transcription factor. It is a homeobox transcription factor that functions as a homodimer or heterodimer with HNF1α. HNF1β plays an important role in organogenesis during embryonic stage, especially of the liver, kidney, and pancreas. Mutations in the HNF1β gene cause maturity-onset diabetes of the young type 5 (MODY5), renal cysts, genital malformations, and pancreas atrophy. Recently, it has been shown that the expression of HNF1β is associated with cancer risk in several tumors, including hepatocellular carcinoma, pancreatic carcinoma, renal cancer, ovarian cancer, endometrial cancer, and prostate cancer. HNF1β also regulates the expression of genes associated with stem/progenitor cells, which indicates that HNF1β may play an important role in stem cell regulation. In this review, we discuss some of the current developments about HNF1β and tumor, the relationship between HNF1β and stem/progenitor cells, and the potential pathogenesis of HNF1β in various tumors.

## Background

Hepatocyte nuclear factors (HNFs) are a group of transcription factors that play important roles in regulating transcription of the liver specific genes. HNFs are expressed predominately in the liver and form a complicated network regulating liver development and hepatocyte differentiation. However, these transcription factors are not restricted to hepatocytes, they are also expressed in many other tissues. Nevertheless, the liver is the only tissue in which a significant number of different HNFs are expressed at the same time [1]. Four major families of HNFs have been described. HNF1α and HNF1β, members of the HNF1 family contain a POU-homeodomain and bind to DNA as homodimers [2]. The HNF3 proteins (HNF3α, β and γ) belong to the forkhead transcription factors and contain a 110 amino acid DNA binding domain [3]. HNF4 is a member of the nuclear hormone receptor family and binds DNA as a homodimer [4]. There are two isoforms of HNF4, HNF4α and HNF4γ, encoded by two separate genes HNF4A and HNF4G in humans [1]. HNF6 contains a bipartite onecut-homeodomain sequence and binds to specific DNA sequences of numerous target gene promoters [5]. Among these HNFs, HNF1β is one of the most important during development and tumorigenesis. In this review, we will focus on HNF1β, which regulates the expression of genes that are expressed in the liver, kidney, and pancreas, and has been identified to cause various human diseases.

The HNF1β gene (TCF2) is located on chromosome 17q12 [6]. The first description of HNF1β mutations associated with disease was in 1997. In humans, heterozygous germline mutations in HNF1β cause maturity-onset diabetes of the young, subtype 5 (MODY5), which is associated with congenital abnormalities, including polycystic kidneys, an abnormal genital tract, and severe pancreatic hypoplasia [7]. HNF1β, also called variant HNF1 (vHNF1) or LFB3, is a homeodomain protein that plays an essential role in the liver-specific expression of many genes during differentiation and development [8]. HNF1β binds to DNA as a homodimer or heterodimer with the related protein HNF1α [9]. The expression ratio of HNF1β is different in each organ. At the adult stage, HNF1β is strongly expressed throughout the biliary system, and in several epithelia organized in tubules, such as the pancreatic exocrine ducts and the kidney tubules [10]. Expression of HNF1β is also seen in the periportal hepatocytes, thymus, genital tract, lung and gut [9, 10]. HNF1β is involved in embryonic development and metabolism of the kidney, pancreas, liver and biliary system. Recent studies have shown that expression of HNF1β is associated with cancer risk in several tumors, and HNF1β plays an important role in tumorigenesis.

## HNF1β status in various tumors

### Hepatobiliary malignancies

HNF1β has been demonstrated to be associated with the risk of hepatocellular carcinoma (HCC). The HNF1 family plays a dominant role in liver-specific transcription. Hepatocyte differentiation is linked to the expression of liver-specific proteins and that the expression patterns are controlled primarily at their transcription levels. It is suggested that HNF1α and HNF1β may play distinct roles in regulating gene expression in differentiation and maturity of hepatocytes. Studies showed that cultured cells derived from differentiated hepatoma cells express HNF1α, whereas cultured dedifferentiated hepatoma cells express HNF1β instead of HNF1α. Analysis of the expression of HNF1α and HNF1β mRNA HCC tissues by RT-PCR assay showed that the ratio of HNF1α/HNF1β mRNA is closely linked to histological differentiation of HCC [11]. The ratio of HNF1α/HNF1β mRNA is higher in well-differentiated cases than in poorly-differentiated and undifferentiated cases. There were more HNF1α than HNF1β transcripts in well-differentiated HCC, but fewer HNF1α than HNF1β transcripts in poorly differentiated HCC. Western blot revealed that the levels of HNF1β protein were similar in well and poorly differentiated HCC, but higher than in the surrounding non-cancerous portions [12]. HNF1β is expressed in hepatic endoderm of foregut during embryonic development and precedes HNF1α expression. In the process of hepatic development and carcinogenesis, members of the HNF1 family regulates the activity of the AFP promoter. Shim et al. examined the expression of alphafetoprotein (AFP), HNF1α, and HNF1β with immunohistochemistry in HCC tumor [13]. The results showed that expression of HNF1β was related to a serum AFP level and AFP expression. Upstream regulation of AFP expression by HNF1β at the transcriptional level may operate specifically during the course of HCC progression following recurrence. The expression of HNF1β in tumor tissue could predict recurrence and HCC-specific death after transplantation [13]. HNF1β also plays an important role in the regulatory network in mouse liver cells. When HNF1β is suppressed by siRNA, the expression level of HNF4α, HNF1α, HNF3 (HNF3α, β and γ), and HNF6 are significantly downregulated [14]. In mouse hepatoma cells, HNF1β regulated many genes. Albumin, α-fetoprotein, insulin-like growth factor binding protein 1 and HNF1 genes were downregulated by RNAi of HNF1β, while alcohol dehydrogenase 2, α1-antitrypsin, α-fibrinogen and apolipo-protein AII genes were upregulated [14]. However, the study of HNF1β and HCC is limited. Thus, further study is still needed to understand the role of HNF1β in hepatocarcinogenesis in HCC and clinical use of HNF1β expression.

HNF1β is expressed in biliary precursor cells and plays a crucial role in extrahepatic biliary development [10]. Biliary tract cancer (BTC) originates from epithelial cells of the hepatic biliary duct system and the gallbladder. Study shows expression of HNF1β is relatively low in BTC, and HNF1β is nonessential in the biliary tract cancers differentiation and maintenance [15].

### Pancreatic cancer

Pancreatic carcinoma is the fourth most common cause of death among cancers. In pancreas, HNF1β is a useful marker to identify clear cell carcinomas, and its overexpression may predict worse survival. Kim et al. [16] found that HNF1β was overexpressed in clear cell carcinomas and in the clear cell components of ductal carcinomas with clear cell features. High expression of HNF1β showed worse survival regardless of morphologic subtype.

### Colorectal cancer

Colorectal cancer (CRC) is the third and fourth most common cancer in females and males. Studies have indicated that the accumulation of genetic and epigenetic alterations could result in the transformation of normal colonic epithelial cells to adenocarcinomas [17]. SILVA et al. investigated the epigenetic changes (DNA methylation) in 24 candidate genes in CRC tumors, and identified five candidate hypermethylated (HM) genes as CRC biomarkers. These genes include RUNX3, PCDH10, SFRP5, IGF2 and HNF1β [17]. Thus, HNF1β may be used as a biomarker for the detection of early-stage of CRC.

### Renal and urinary tract cancer

Several studies suggest a role of HNF1β in tumor formation and various kinds of renal disease. Renal cell carcinoma (RCC) accounts for more than 80 % of kidney tumors. Studies found that HNF1β expression was reduced in tumor tissue compared with normal kidney tissue. HNF1β is essential for the maintenance of well-ordered renal tissue growth, and downregulation or loss of expression is correlated with malignant transformation and dedifferentiation. HNF1β is a prognostic factor and a potential tumor suppressor, and could be a potential therapeutic target for RCC [18]. Chromophobe renal cell carcinoma (ChRCC) is a rare type of kidney cancer and accounts for 5 % of all kidney cancer. Lack of HNF1β expression plays an important role in the pathogenesis of ChRCC, and may serve as a good diagnostic maker [19]. Rebouissou et al. screened for HNF1β inactivation in 35 renal neoplasms and found biallelic HNF1β inactivation in 2 chromophobe renal carcinomas by association of a germline mutation and a somatic gene deletion. This finding suggested that germline mutations of HNF1β may predispose to renal tumors and proposed that HNF1β may functions as a tumor suppressor gene in ChRCC through a PKHD1 expression control [20]. Gad et al. studied 46 cases of ChRCC, but no mutations were identified in all coding exons of the HNF1β gene. They found that mutations in BHD and TP53 genes but not in HNF1β gene were detected in a large series of sporadic ChRCC [21]. Papillary renal cell carcinomas make up about 15 % of renal cell tumors in surgical series. The HNF1β protein is expressed only in differentiating tubules of fetal kidney and overexpressed in adult tumors of embryonal origin, such as papillary RCCs. The gain and overexpression of the HNF1β gene is associated with the delayed tubular differentiation in precursor lesions [22]. Clear cell adenocarcinoma (CCA) of the urinary tract is a rare tumor that is histologically similar to CCA of the female genital tract [23]. Brimo et al. reported that HNF1β was a useful marker in differentiating clear cell adenocarcinomas of the bladder/urethra from invasive high-grade transitional/urothelial carcinoma and other types of bladder adenocarcinomas and to a lesser extent from nephrogenic adenomas [8].

### Prostate cancer

Prostate cancer is one of the most common male malignancies. Recently, a large study has shown that an HNF1β sequence variant confers an increased prostate cancer risk. The first report of HNF1β associated with prostate cancer risk was in a genome-wide association study (GWAS) searching for sequence variants in 1501 Icelandic men with prostate cancer and 11,290 controls, and the variant, rs4430796, in HNF1β at chromosome 17q12 was the earliest loci to be discovered for prostate cancer [6]. It was later replicated in two GWAS in the UK and the United States [24, 25]. Then a second independent variant, rs11649743, located at chromosome 17q12 and separated by a recombination hotspot from the first variant, was subsequently found to be associated with risk [26]. A large-scale fine mapping study of a region on 17q12 associated with prostate cancer confirmed the previously established signals and found evidence that additional variants contribute to the risk of prostate cancer [27]. In this study, the best model for prostate cancer risk in the HNF1β region included five SNPs, rs4430796, rs7405696, rs4794758, rs1016990 and rs3094509, and these SNPs together capture more of the risk associated with this region. Zhang et al. explored the loci associated with prostate risk in a Northern Chinese population and indicated that AG on HNF1β (rs4430796, A) could be associated with PSA increase [28]. Hu et al. identified 12 prostate cancer risk genes potentially connected and related to HNF1β. Six of them, BAG1, ERBB4, ESR1, HSPD1, NR4A1, and PIK3CG, were found to participate in the KEGG pathways. The results indicated that the prostate cancer risk role of HNF1β could possibly be associated with modulating the relationships between androgenic hormone and prostate cancer [29].

### Ovarian and endometrial cancer

HNF1β was identified from large-scale gene expression studies as being a useful marker of ovarian and uterine clear cell carcinomas. Clear cell carcinoma (CCC) of the ovary has the worst prognosis of all of the epithelial ovarian cancers. A study has demonstrated that the expression of HNF1β is significantly upregulated in ovarian CCC cell lines, while non-CCC ovarian cancer cell lines rarely express this protein, also, reduction of HNF1β induced apoptotic cell death in ovarian CCC cell lines [30]. HNF1β would be not only an excellent CCC-specific molecular marker but also a molecular target for the therapy of ovarian CCC. Yamamoto et al. found that the incidence of HNF1β immunoreactivity differed significantly between CCCs and other histology in both the ovary and the endometrium, which suggested that HNF1β would be an excellent marker for distinguishing CCCs from other lesions in both the ovary and the endometrium [31]. It was demonstrated by Kao et al. that the overexpression of HNF1β is specific for ovarian CCC among ovarian carcinomas [32]. The HNF1β gene plays an important role in the biology of ovarian CCC. Knockdown of HNF1β in ovarian CCC cells resulted in a significant increase in proliferation, while overexpression of HNF1β in the serous ovarian cancer cell line caused cell growth to be significantly decreased [33]. Downregulation of HNF1β could contribute to drug resistance in ovarian cancer and that HNF1β may perform its drug resistance-related functions through four pathways including ErbB signaling, focal adhesion, apoptosis and p53 signaling [34]. Terasawa et al. observed for the first time that HNF1β gene is a target for epigenetic inactivation in ovarian cancer. HNF1β was methylated in 53 % of ovarian cancer cell lines and in 26 % of primary ovarian cancers (especially in the non-clear cell types), resulting in loss of the gene’s expression. Restoration of HNF1β expression induced expression of HNF4α, a transcriptional target of HNF1β, indicating that epigenetic silencing of HNF1β leads to alteration of the hepatocyte nuclear factor network in tumours [35]. Shen et al. found a differential effect of HNF1β on the serous and clear cell subtypes of ovarian cancer, a loss-of-function role in serous and a gain-of function role in clear cell ovarian cancers, and variants in this gene differentially affect genetic susceptibility to these subtypes [36].

Endometrial cancer is the most common gynecological cancer in developed countries. A genome-wide association study identified single nucleotide polymorphisms in HNF1β associated with endometrial cancer risk in women of European background [37]. SNP rs4430796 was identified as an endometrial cancer susceptibility locus close to HNF1β on chromosome 17q. Setiawan et al. provided additional evidence that HNF1β is involved in endometrial cancer etiology [38]. The HNF1β SNPs (rs4430796 and rs7501939) were associated with endometrial cancer risk in two independent studies and that the associations were observed across multiple racial/ethnic groups [38]. A recent genome-wide association study replicated previously identified associations with genetic markers near the HNF1β locus in a multiethnic population from nine studies, but no novel variants reached genome-wide significance [39]. In endometrial cancer, different from ovarian clear cell carcinoma, HNF1β should be used with caution as a diagnostic marker because of its lack of specificity [40].

## The potential pathogenic mechanisms of HNF1β in cancer

### Epigenetic processes and epigenetic changes

The aberrant expression of HNF1β in tumors is associated with epigenetic processes and epigenetic changes. In humans, one of the epigenetic mechanisms that regulate expression of genes is methylation of the clusters of CpG dinucleotides, called CpG islands. A probable mechanism of aberrant up-regulation of HNF1β in ovarian clear cell carcinoma is hypomethylation of the HNF1β CpG island [35]. Terasawa et al. reported that methylation of the HNF1β CpG island was rare in ovarian CCC, but common in non-CCC ovarian cancers or various cancer cell lines [35]. Hypomethylation of the HNF1β CpG island probably participates in the up-regulation of HNF1β in ovarian CCC. Epigenetic inactivation of HNF1β is also seen in colorectal, gastric, and pancreatic cancer cell lines, suggesting involvement of epigenetic inactivation of HNF1β in tumorigenesis [41]. HNF1β mutations are known to affect expression of downstream genes such as HNF4α, PKHD1 and UMOD [20]. HNF4α is upregulated by inducing HNF1β expression, which suggests alterations in the hepatocyte nuclear factor network can be reversed by inducing HNF1β through demethylation of the gene [35]. Another potential contributors to the HNF1β overexpression are histone acetylation and gene amplification. Methylation was associated with histone deacetylation, and when treating cells with histone deacetylase inhibitor combined with methyltransferase inhibitor, HNF1β expression was synergistically induced [35]. Studies showed that homeobox genes, such as the TLX1, HOXB13, and HNF1B genes methylation play a critical role in the insurgence and/or progression of breast cancer [42]. In Sporadic colorectal cancer (CRC), hypermethylated gene HNF1β was identified, which may be a useful epigenetic marker for non-invasive CRC screening [17].

### The HNF1β target genes

The pathway with which HNF1β is involved in cancer is less understood. HNF1β has been identified as one of the most highly overexpressed genes in ovarian CCC. In ovarian CCC cell lines, one of the most markedly up-regulated genes was osteopontin (OPN), which is probably a direct target gene of HNF1β, since OPN contains functional HNF1β binding sites in the promoter region [43]. It was reported that OPN expression is elevated in ovarian CCC and is closely associated with HNF1β overexpression. HNF1β is likely to participate in OPN up-regulation in CCC [44]. OPN has been recognized to play important roles in the process of tumorigenesis. OPN binds to several cell surface receptors and induces several signal transduction pathways, which contribute to tumorigenesis by the inhibition of apoptosis or the activation of matrix-degrading proteases [45]. The reduction of HNF1β might have caused a reduction of OPN, followed by an increase in apoptotic activity. Data showed that checkpoint kinase (Chk) 1 protein is persistently activated in the HNF1β-overexpressing CCC cells [46]. Chk1 might function in the cellular survival pathways that enhance DNA damage repair, thereby granting chemoresistance. Inhibition of Chk1 selectively abrogates the repair of damaged DNA, sensitizes cancer cells to radiotherapy, or increases cancer cell death in the presence of p53 mutations [47]. The Chk1 inhibitor might be a novel target for developing cancer therapeutics in the HNF1β positive cells [46]. Pathway enrichment analysis of 36 genes which co-occurred with HNF1β, ovarian cancer and drug resistance was performed by Li et al. [34]. Four pathways including ErbB signaling, focal adhesion, apoptosis and p53 signaling were enriched, suggesting that HNF1β may contribute to drug resistance in ovarian cancer via those pathways. HNF1β may also play a critical role in a cytoprotective effect against forthcoming oxidative stress. HNF1β may upregulate OGG1 gene expression to counteract ROS mediated mitochondrial dysfunction [48]. OGG1 also known as 8-oxoguanine glycosylase is a DNA glycosylase enzyme involved in base excision repair. Senkel et al. established a human embryonic kidney cell line (HEK293) expressing HNF1β and identified 25 HNF1β-regulated genes. Eight of the 25 genes were significantly up-regulated in ovarian CCC compared to the other ovarian cancer types. The genes SPP1, DPP4, SAH, RBPMS, CD24, NID2, LAMB1, RHOB and SOX9 are deregulated in ovary CCC due to the overexpression of HNF1β [43]. The genes dipeptidyl peptidase 4 (DPP4), and osteopontin (SPP1) are most likely direct target genes, as they contain functional HNF1 binding sites in their promoter region. DPP4 plays a major role in glucose metabolism and appears to work as a suppressor in the development of cancer [49]. RBPMS (RNA binding protein multiple splicing) may be an HNF1β target involved in kidney formation. CD24 is a cell adhesion molecule that is identified as a progenitor marker. NID2 encodes a member of basement membrane proteins that control a large number of cellular activities [50]. LAMB1 (Laminin, beta 1) is a member of noncollagenous constituent of basement members. Sox9 [sex-determining region Y (SRY)-box 9 protein] plays critical roles during embryogenesis. Sox9 is required for development, differentiation, and lineage commitment in various tissues. Studies showed that 22 of 54 genes highly up-regulated in ovarian CCC were involved in downstream targets of HNF1β. These genes include GLRX, GPx3, TST, SOD2, NNMT, ANXA4, UGT1A1, DPPIV, ACE2, Collectrin, TFPI2, MAP3K5/ASK1, Octamer4, PAX8, G6Pase, GK, GLUT2, ALDOB, OPN, and FXYD2 [51]. Genes reported to be upregulated by HNF1β include NNMT, ANXA4, UGT1A1, FXYD2, TFPI2, MAP3K5/ASK1, G6Pase, GK, GLUT2, and mTOR [51]. GLRX (Glutaredoxin), GPx3 (glutathione peroxidase 3), TST (thiosulfate sulfurtransferase), SOD2 (superoxide dismutase2), NNMT (Nicotinamide N-methyltransferase), ANXA4 (Annexin A4), and UGT1A1 (UDP-glycosyltransferase 1 family polypeptide A1) are responsible for drug metabolism and liver detoxification. DPPIV (Dipeptidyl peptidase IV), ACE2 (Angiotensin converting enzyme2), Collectrin, and TFPI2 (Tissue factor pathway inhibitor 2) are proteases involved in oxidative stress. MAP3K5/ASK1 and mTOR are related to signal transduction pathways. Octamer4 (Octamer-binding transcription factor 4) and PAX8 (Paired box gene 8) are transcription factors involved in oxidative stress. G6Pase (Glucose-6-phosphatase), GK (Glucokinase), GLUT2 (Glucose transporter type 2), and ALDOB (Aldolase B) are related to metabolism. FXYD2 (domain-containing ion transport regulator 2) is known as the gamma subunit of the Na, K-ATPase. HNF1β also regulates the expression of CD44v9, which binds several target molecules and specifically regulates cell functions, including migration, growth, survival, anti-apoptosis, immune response and redox status [52]. In human hepatoma cells, HNF4α has been suggested to be directly regulated by HNF1β [53]. The HNF1β-dependent pathway might provide new insights into regulation of glycogen synthesis, detoxification and resistance to anticancer agents.

### The signaling related to stem cells

HNF1β has been described as a key regulator of biliary development and is partially regulated by Notch signaling [54]. The Notch pathway has been implicated in the regulation of self-renewal of adult stem cells and differentiation of precursors along a specific cell lineage, in normal embryonic development and organogenesis [15]. Studies showed that HNF1β expression is regulated by Notch2 [54]. Notch2 is a member of the notch family, and it is used to isolate, identify and localise pancreatic cancer stem-like cells. Studies showed that Notch2^+^ cells in human pancreatic cancer Bxpc-3 and Panc-1 cells have the properties of cancer stem cells, with strong tumourigenic ability [55]. Gene expression microarray data demonstrated that Notch2 is one of the most upregulated genes in a cancer stem cell-like population [56]. Moreover, aberrant Notch2 signaling induces the formation of human liver cancers with HpSC features [57].

## HNF1β and stem/progenitor cells

Genes and pathways regulating stem and progenitor cells are increasingly recognized in tumorigenesis. Tumors may often originate from the transformation of normal stem cells, since similar signalling pathways may regulate self-renewal in stem cells and cancer cells. According to the cancer stem cell (CSC) hypothesis, a few CSC with self-renewal and multipotentiality differentiation exist in tumors, and these cells can create heterogeneity in a tumor through abnormal proliferation and differentiation. In HEK293 cells and ovary CCC, HNF1β activates the CD24 gene, a cell surface protein that has recently been identified as a marker of the renal progenitor population in the uninduced metanephric mesenchyme [43]. CD24 is highly expressed in many human cancers [58], and is often used to identify and enrich CSCs in cancers such as ovarian and pancreatic cancer. High expression of CD24 is involved in tumor progression and metastasis. SOX9 was down-regulated upon HNF1β overexpression in HEK293 cells. SOX9 is overexpressed in a wide range of human cancers, particularly, in tissues where it plays critical roles in their development and in stem/progenitor cells [59]. SOX9 might contribute to carcinogenesis through effects on stem cells. The HNF1β target gene osteopontin is also shown to be expressed in progenitor cells. OPN is overexpressed by liver progenitors in humans and mice. OPN upregulation during liver injury is a conserved repair response, and influences liver progenitor cell function [60]. A high OPN expression level is associated with poor prognosis and metastasis in several cancer patients. The expression of CD44 variant forms is regulated by several molecules including OPN. CD44 interacts with osteopontin and regulates its cellular functions leading to tumor progression. HNF1β also regulates the expression of CD44v9. CD44 is a multifunctional class I transmembrane glycoprotein with a variety of functions including participation in cell adhesion and migration as well as modulation of cell–matrix interactions [61]. The majority of cancer cell lines express high levels of CD44. CD44 is one of the cell surface markers associated with cancer stem cells in several types of tumor, including breast, prostate, pancreas, ovarian, and colorectal cancers [52, 58]. Overexpression of CD44 indicated bad clinical features and poor prognosis.

In liver development, HNF1β is involved in the hepatobiliary specification of hepatoblasts to cholangiocytes, and it is strongly expressed throughout the embryonic and adult biliary epithelium. Immunostaining of patients with advanced alcoholic liver disease showed that ductular reaction cells were positive for HNF1β, whereas HNF1β was not expressed in mature hepatocytes [62]. It is demonstrate that HNF1β is expressed in liver progenitor cells (LPC), and may also play a role in the maintenance of the hepatobiliary cell phenotype. Lineage tracing demonstrated that HNF1β+ cells give rise to the expansion of cells with a LPC phenotype and to periportal hepatocytes after liver injury [62]. Yu et al. reported that the induced hepatic stem cells (iHepSCs) can be directly induced from mouse embryonic fibroblasts by overexpressing two key transcription factors, HNF1β and Foxa3 [63]. HNF1β has a critical role in the induction of iHepSCs. Pancreatic multipotent progenitor cells (MPCs) produce acinar, endocrine and duct cells during organogenesis [64]. HNF1β has been identified in these pancreatic progenitor cells before differentiation into endocrine or exocrine cells [65, 66]. Study showed that Sox9 directly bound to three genes, including HNF1β, HNF6, Foxa2, in vitro and in intact cells, and regulated their expression. In turn, both Foxa2 and HNF1β regulated Sox9 expression, demonstrating feedback circuits between these genes. Foxa2 (forkhead box protein A2), also known as HNF3β, is a member of the forkhead class of DNA-binding proteins. Foxa2 play an important role in the regulation of metabolism and in the differentiation of the pancreas and liver. HNF6 is correlated to cell proliferation, differentiation and organogenesis, hepatic metabolism [5]. HNF1β, HNF6, and Foxa2 are simultaneously expressed in several regions in the early endoderm, including the developing gut and liver, and persist in the duct cells of the adult pancreas [65]. Transient knockdown of Sox9 levels by nearly 50 % resulted in a 50 % increase in the expression of HNF1β [65]. HNF1β which is expressed before HNF6 in the endoderm, was found to be critical for HNF6 expression. HNF6 controls the initiation of the expression of pancreatic and duodenal homeobox 1 (Pdx1), the earliest marker of pancreatic precursor cells [67]. Therefore, the sequential activation of HNF1β, HNF6, and Pdx1 in the endoderm appears to control the generation of pancreatic precursors. Studies showed that Pdx1 contributed to the specification of pancreatic endocrine progenitors by participating in the Hnf6, Sox9, Hnf1β, Foxa2 transcription factor crossregulatory network and by regulating Ngn3 directly [68]. Ngn3 (neurogenin3) plays an important role in pancreatic development and the differentiation of endocrine cells. Ngn3 is also one of the pancreatic progenitor markers. Because HNF1β is required for Ptf1a expression in the developing pancreatic bud, Ptf1a may also be downstream of HNF1β when generating pancreatic precursors in the endoderm [67]. Ptf1a (pancreas transcription factor 1a) is expressed in early bud pancreatic multipotent progenitor cells, with an instructive role in distinguishing pancreatic fate from the adjacent organs [64]. All these studies showed that HNF1β is closely related to stem/progenitor cells.

## Conclusions

HNF1β plays an important role in tumorigenesis of various organs. High expression of HNF1β showed worse survival in both pancreatic carcinoma and hepatocelullar carcinoma. HNF1β expression is reduced in renal cell carcinoma compared with normal kidney tissue. HNF1β may functions as a tumor suppressor gene in renal cell carcinoma. In prostate cancer, the studies of HNF1β focus mainly on the single nucleotide polymorphisms. Genome-wide association studies have discovered at least 30 susceptibility loci for prostate cancer. It is demonstrated that HNF1β would be an excellent marker for distinguishing CCCs from other lesions in both the ovary and the endometrium. In ovarian cancer, downregulation of HNF1β may contribute to drug resistance, thus estoration of HNF1β function could be a therapeutic approach. Many genetic and epigenetic alterations of HNF1β gene as well as several genetic networks and signaling pathways that are considered to be involved in the development and progression of tumor. The details of the regulatory pathways and their mechanisms are still under investigation. Studies also showed that HNF1β is closely associated with stem/progenitor cells. However, the potential pathogenic mechanisms of HNF1β in cancer and regulatory mechanisms in stem cells are still less understood. Thus, further study for HNF1β with tumor and stem cell is still needed.
